# Development of a Loop-Mediated Isothermal Amplification Assay for Rapid Detection of *Trichosporon asahii* in Experimental and Clinical Samples

**DOI:** 10.1155/2015/732573

**Published:** 2015-01-27

**Authors:** Jianfeng Zhou, Yong Liao, Haitao Li, Xuelian Lu, Xiufeng Han, Yanli Tian, Shanshan Chen, Rongya Yang

**Affiliations:** ^1^Department of Dermatology, General Hospital of Beijing Military Command of PLA, No.5, Nanmencang, Dongcheng District, Beijing 100010, China; ^2^The Clinical Medical College in the Beijing Military Region of Second Military Medical University of PLA, Beijing 100700, China

## Abstract

Invasive trichosporonosis is a deep mycosis found mainly in immunocompromised hosts, and the major pathogen is *Trichosporon asahii*. We detected the species-specific intergenic spacers (IGS) of rRNA gene of *T. asahii* using a loop-mediated isothermal amplification (LAMP) assay in 15 isolates with 3 different visualization methods, including SYBR green detection, gel electrophoresis, and turbidimetric methods. The LAMP assay displayed superior rapidity to other traditional methods in the detection time; that is, only 1 h was needed for detection and identification of the pathogen DNA. Furthermore, the detection limit of the LAMP assay was more sensitive than the PCR assay. We also successfully detect the presence of *T. asahii* in samples from experimentally infected mice and samples from patients with invasive trichosporonosis caused by *T. asahii*, suggesting that this method may become useful in clinical applications in the near future.

## 1. Introduction

*Trichosporon asahii* (*T. asahii*) is a type of anamorphic basidiomycetous yeast that exists widely in natural conditions [1, 2]. It can cause many human diseases, such as superficial infections [2, 3], summer-type hypersensitivity pneumonitis [4, 5], and invasive infections [6–9].* T. asahii *belongs to the genus* Trichosporon*, which contains approximately 50 species of basidiomycetous yeasts, which have been detected in the skin and in the gastrointestinal, respiratory, and urinary tracts of humans and can cause superficial infections in immunocompetent individuals, such as white piedra infections of the hair shaft.* Trichosporon* species, especially* T. asahii*, can also cause invasive trichosporonosis in immunocompromised individuals and critically ill patients in the ICU, resulting in high mortality rates (50–80%) [10–13]. The genus* Trichosporon* was the third most commonly isolated non-*Candida* yeast from clinical specimens in the ARTEMIS DISK global antifungal surveillance study [14] and was the second most common cause of yeast fungemia in patients with hematological malignancies, after* Candida* species [10, 11].

Early detection of the agent of invasive trichosporonosis is critical for prompt and effective treatment [10, 11]; this is difficult in resource-limited settings without essential diagnostic facilities [15]. At present, a proven diagnosis of invasive trichosporonosis depends on the sterile culture of fungus from body fluids or other related tissue samples and subsequent identification by microscope and biochemical assay [10, 15, 16]. Furthermore,* Trichosporon asahii*, the most common cause of disseminated trichosporonosis, may be easily mistaken for* Candida *spp. and other* trichosponron strains* in culture, especially if mixed yeast or mold species are recovered [17, 18]. Based on morphological characteristics and biochemical profiling, methods for identifying* Trichosporon* isolates at the species level are time consuming, requiring specialist training and appropriate laboratory facilities, and may yield inconsistent results [17].

Molecular tests for rapid and specific identification of the medically relevant* Trichosporon* species were developed [19]. The technique incorporates PCR primers designed from DNA sequences within the conserved regions of the rRNA exons and amplifies variable sequences found in the internal transcribed spacer (ITS) and intergenic spacer (IGS) regions of the tandemly repeated 18S, 5.8S, and 26S fungal rRNA and the D1/D2 region of the 26S rRNA gene. Xiao et al. applied reverse line blot (RLB) hybridization and rolling circle amplification (RCA) assays based on the ITS region for the identification of* Trichosporon* species [20]. However, there is limited information about the clinical molecular detection and identification of* T. asahii *strains to discriminate it from other clinically important fungal pathogens.

As a result of the rapid development of molecular biology methods, a new identification technique based on polymerase chain reaction (PCR) technology has been widely described. Notomi et al. reported a new DNA loop-mediated amplification (LAMP) method [21]. It can be used to amplify specific target DNA sequences with high sensitivity, and the amplification can be obtained in approximately 60 min with four specific primers and strand displacement DNA polymerase in isothermal conditions (approximately 65°C), eliminating the need for a thermal cycler. The LAMP assay has been reported to be highly specific, sensitive, rapid, and cost-effective [22, 23]. In addition, the LAMP assay could be carried out in a typical laboratory using a water bath or a heating block. It is a potentially valuable means for clinical sample testing. To facilitate the rapid and inexpensive molecular diagnosis of* T. asahii*, we developed a LAMP assay targeting the IGS1 region and evaluate this assay by using* T. asahii*-positive clinical samples.

## 2. Materials and Methods

### 2.1. Strains and Culture Conditions

In total, 15* Trichosporon asahii* strains, 14 other* trichosponron strains*, and 23 reference strains of related melanized fungi (Table 1) were used to establish the specificity of the LAMP assay.

### 2.2. DNA Extraction

For DNA extraction, two loopfuls of cultures grown on PDA agar (Oxoid Ltd., Basingstoke, Hampshire, England) for 2 to 5 days at 25°C were suspended in 500 *μ*L of lysing buffer (50 mM Tris, 250 mM NaCl, 50 mM EDTA, 0.3% sodium dodecyl sulfate (SDS) (pH 8.0)) plus the equivalent of a 200 *μ*L volume of 425 to 600 um diameter glass beads (Sigma). After being vortexed for 2 min, DNA was isolated using the DNAsecure Plant Kit (TIANGEN) according to the manufacturer's protocol. We used a NanoDrop 2000 (Thermo Scientific, Rockford, IL) to quantify the DNA extracted from the CBS2479 strain (9.4 ng/*μ*L) and then calculated the copy numbers (3.56 × 10^6^ copies/*μ*L) from the 24,271,268 kb full-length genome. DNA was serially diluted 1 : 10 up to 10^−6^-fold dilutions (9.4 ng/*μ*L to 9.4 fg/*μ*L), and 1 *μ*L of each serial dilution was used as a template in the LAMP systems. DNA solutions could be kept for several months at −20°C without noticeable degradation.

### 2.3. Design of LAMP Primers

Five sets of four species-specific LAMP primers were designed based on the* T. asahii* identifiable target, the rRNA gene IGS1 region. The LAMP primers were designed using the PRIMEREXPLORER V4 software program (http://primerexplorer.jp). A forward inner primer (FIP) consisted of the complementary sequence of F1 (F1c) and F2, and a backward inner primer (BIP) consisted of B1c and B2. The outer primers F3 and B3 were required for initiation of the LAMP reaction. The sequences of each primer are shown in Table 2.

### 2.4. LAMP Reaction

The reaction mixture concentrations of each component were as follows: 20 mM Tris-HCl (pH 8.8), 10 mM KCl, 10 mM (NH_4_)_2_SO_4_, 0.1% Triton X-100, 0.8 M betaine, 8 mM MgSO_4_, 1.4 mM dNTP, 8 U Bst DNA polymerase, 40 pmol FIP and BIP, 5 pmol F3 and B3, and 20 pmol LB and LF, with 1 *μ*L of crude DNA extract as the template. A reaction mixture volume of 25 *μ*L was incubated at a constant temperature of 65°C for approximately 60 min. Double distilled water was used as a negative control.

### 2.5. Analysis of LAMP Products

Real-time turbidity caused by the accumulation of magnesium pyrophosphate was monitored spectrophotometrically at 650 nm with the LA-320C Loopamp Realtime Turbidimeter (Eiken Chemical Co., Ltd., Tochigi, Japan). The results were analyzed with the LA-320C software package. The product was electrophoresed on an agarose gel in Tris-acetate-EDTA (TAE) buffer followed by staining with ethidium bromide. Amplified products were sequenced to show that they matched the expected nucleotide sequences. Positive amplification was shown by the specific ladder-like pattern on a UV transilluminator at 320 nm. For visualization of the positive reaction, a fluorescent detection reagent (FD; Eiken Chemical Co., Ltd. Tokyo, Japan) was added to the reaction mixture and a change of color was observed visually (transparent to green color), while those remaining orange were considered negative.

After the LAMP reactions, 1 *μ*L of each product was used for 1% agarose gel electrophoresis (100 V, constant for 40 min). A Gel Doc XR+ imaging system (Bio-Rad, Hercules, CA) was used to observe the band patterns. The samples were considered positive if they showed a characteristic ladder-like band pattern.

### 2.6. PCR Assay

PCR assay was performed to compare the sensitivity with the established LAMP methods. The outer primers (F3 and B3) of LAMP assay were used for PCR assay. The 25 *μ*L volume reaction mixture contained 1× PCR buffer (10 mM Tris–HCl (pH 8.3), 50 mM KCl, 1.5 mM MgCl_2_), 0.2 mM each dNTP, 0.2 *μ*M each primer (F3 and B3), 1 U Taq DNA polymerase (TaKaRa Biotechnology Co., Ltd., Dalian, China), and 5 *μ*L template DNA. Each reaction was initially denatured at 95°C for 5 min; followed by 35 cycles of 94°C for 30 s, 55°C for 30 s, and 72°C for 30 s; and a final extension at 72°C for 3 min using a C1000 Thermal cycler (Bio-Rad Laboratories, Hercules, CA, USA). PCR products were separated on 1% (w/v) agarose gel in 1× TAE stained with EB and photographed using a GelDoc XR+ Imager (Bio-Rad, Hercules, CA).

### 2.7. Experimental Infection of* T. asahii*

Ten male BALB/c mice, 6–8 weeks old, were purchased from the laboratory animal research center of the China Academy of Chinese Medical Sciences and were housed in two cages, containing 5 mice each. Mice were provided with food and water. The mice in the infection model group received two intraperitoneal injections of 150 mg/kg cyclophosphamide (Jiangsu Hengrui Medicine Co., Ltd, Jiangsu, China) in sterile saline 2 days before and 1 day after fungal inoculation. On the day of infection, the mice in infection group were intravenously inoculated with 1 × 10^7^ cfu/mouse of* T. asahii* in a 0.2 mL volume by injection into the lateral tail vein, and the mice in control group were not treated.

### 2.8. Evaluation of the LAMP Assay Using Experimental Animal and Clinical Samples

To evaluate the LAMP assay, blood, urine, and peritoneal irrigation fluid were collected from 5 experimentally infected mice and 5 control mice. The animal samples were tested by LAMP assay and conventional culture in parallel, and the human samples had been tested by API 20 C AUX systems (bioMérieux) before LAMP. All samples (Table 3) were transferred to Eppendorf tubes and centrifuged at 12,000 ×g for 5 min. The cells were resuspended in 100 *μ*L of cell lysis buffer and boiled at 100°C for 5 min, and the supernatant DNA was extracted with DEXPATs (TaKaRa Biomedical, Inc., Otsu, Japan) according to the manufacturer's instructions.

## 3. Results

### 3.1. Primer and Temperature Selection for the LAMP Reaction

In this study, we designed 5 sets of primers for LAMP amplification. Among these primers, those for IGS-LAMP had the highest amplification rates and the shortest peak appearance times. We concluded that the TA-19 LAMP reaction primer combination is the best. To optimize the LAMP reaction temperature, we conducted the procedures under different conditions. LAMP assays had been performed under isothermal conditions between 59°C and 66°C. Considering the larger amounts of DNA amplicons and the optimal temperature for Bst DNA polymerase activity, we choose 65°C as the final reaction temperature (data does not show).

### 3.2. Specificity of the LAMP Reaction

**Figure 1:**
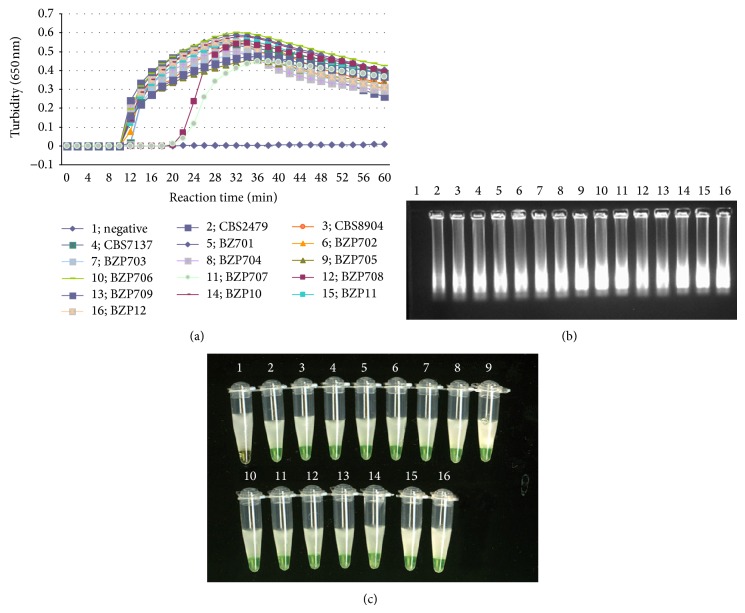
Specificity of LAMP detection for different* T. asahii* strains. Lane 1: negative control; lane 2: standard* T. asahii* CBS2479; lane 3:* T. asahii* CBS8904; lane 4:* T. asahii* CBS7137; lane 5:* T. asahii* BZ701; lane 6:* T. asahii* BZ702; lane 7:* T. asahii* BZ703; lane 8:* T. asahii* BZ704; lane 9:* T. asahii* BZ705; lane 10:* T. asahii* BZ706; lane 11:* T. asahii* BZ707; lane 12:* T. asahii* BZ708; lane 13:* T. asahii* BZ709; lane 14:* T. asahii* BZ710; lane 15:* T. asahii* BZ901; lane 16:* T. asahii* BZ902. (a) Specificity of LAMP assay detected by real-time measurement of turbidity (LA-320C). A positive reaction was defined as a threshold value of >0.1 within 80 min. A positive reaction was observed in all* T. asahii *isolates, whereas the negative controls showed no increase in turbidity. (b) Electrophoresis (1%) applied to loop-mediated amplification products from different* T. asahii *strains. The positive reaction was seen as a ladder-like pattern on 1% agarose gel electrophoresis analysis. (c) The specificity of LAMP for* T. asahii* detection by direct observation. A green colour was observed using the naked eye in the tube which contained* T. asahii*, whereas the negative controls remained light orange after the reaction.

**Figure 2:**
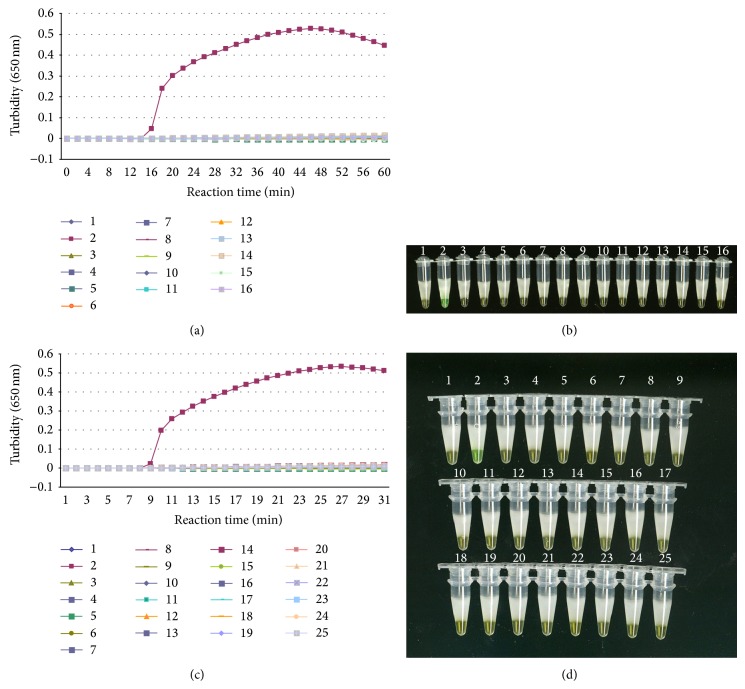
Specificity of LAMP detection for* T. asahii*. (a), (b) Lane 1: negative control; lane 2:* T. asahii *isolate CBS2479; lane 3:* Trichosporon domesticum*; lane 4:* Trichosporon dermatis*; lane 5:* Trichosporon jirovecii*; lane 6:* Trichosporon mucoides*; lane 7:* Trichosporon asteroides*; lane 8:* Trichosporon coremiiforme*; lane 9:* Trichosporon inkin*; lane 10:* Trichosporon japonicum*; lane 11:* Trichosporon lactis*; lane 12:* Trichosporon ovoides*; lane 13:* Trichosporon dohaense*; lane 14:* Trichosporon faecale*; lane 15:* Trichosporon debeurumanianum*; lane 16:* Trichosporon montevideense*. (a) Specificity of the LAMP assay monitored by real-time measurement of turbidity (LA-320C). Positive reaction was observed in* T. asahii*, whereas none of other* Trichosporon* spp. showed turbidity increases. (b) The specificity of LAMP for* T. asahii *detection by naked eye detection. Green colour was observed using the naked eye in the tube which contained* T. asahii*, whereas others remained light orange after the reaction. (c), (d) Lane 1: negative control, lane 2:* T. asahii *isolate CBS2479; lane 3:* Cryptococcus gattii*; lane 4:* Cryptococcus laurentii*; lane 5:* Cryptococcus luteolus*; lane 6:* Cryptococcus neoformans var. grubii*; lane 7:* Cryptococcus neoformans var. neoformans*; lane 8:* Cryptococcus podzolicus*; lane 9:* Cryptococcus podzolicus*; lane 10:* Pichia pastoris*; lane 11:* Candida albicans*; lane 12:* Candida glabrata;* lane 13:* Candida krusei*; lane 14:* Candida parapsilosis*; lane 15:* Candida parapsilosis;* lane 16:* Candida stellata*; lane 17:* Candida tropicalis*; lane 18:* Debaryomyces hansenii*; lane 19:* Fonsecaea pedrosoi*; lane 20:* Exophiala dermatitidis*; lane 21:* Filobasidiella neoformans var. neoformans*; lane 22:* Sporothrix schenckii*; lane 23:* Acremonium chrysogenum*; lane 24:* Aspergillus fumigatus*; lane 25: Aureobasidium pullulans. (c) Specificity of the LAMP assay monitored by real-time measurement of turbidity (LA-320C). Positive reaction was observed in* T. asahii*, whereas none of other non-*Trichosporon* fungal strains showed turbidity increases. (d) The specificity of LAMP for* T. asahii *detection by naked eye detection. Light blue colour was observed using the naked eye in the tube which contained* T. asahii*, whereas others remained light orange after the reaction.

To determine specificity of the primers, 51 isolates were also subjected to the LAMP assay. A Loopamp Realtime turbidimeter (LA-320C) was employed to detect the LAMP products in the LAMP assays. As shown in Figure 1(a), LAMP products were amplified only from DNA samples of* T. asahii*, and the typical time threshold curve indicating turbidity values was obtained, whereas other related* Trichosporon* strains (Figure 2(a)) and other fungal strains (Figure 2(c)) did not show increases in turbidity. No false positive amplification was observed, indicating the high specificity of the established LAMP assays. Amplification was completed within 1 h isothermally at 65°C in a water bath. The products of the LAMP reaction could be detected by electrophoresis on 1% agarose gels and showed ladder-like patterns (Figure 1(b)). The products were also visible in Eppendorf vials. Positive reactions underwent a color change to green, whereas negative reactions remained light orange (Figures 1(c), 2(b), and 2(d)).

### 3.3. Sensitivity of the LAMP Reaction

**Figure 3:**
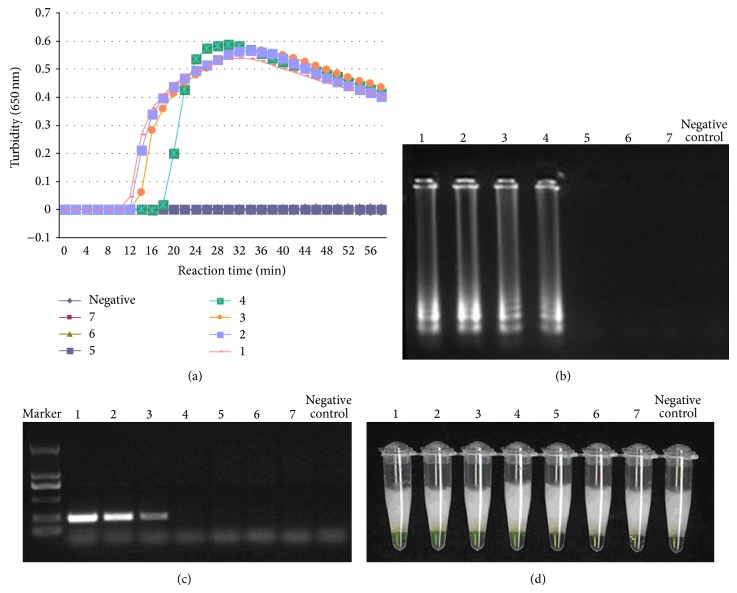
Sensitivity of LAMP method using serially diluted genomic DNA (1, 3.56 ∗ 10^6^ copies, 2, 3.56 ∗ 10^5^ copies, 3, 3.56 ∗ 10^4^ copies, 4, 3.56 ∗ 10^3^ copies, 5, 3.56 ∗ 10^2^ copies, 6, 3.56 ∗ 10^1^ copies, 7, 3.56 copies) with* T. asahii* CBS2479 as template. The detection limit for the assay was 3.56 ∗ 10^3^ genomic copies. (a) Sensitivity of the LAMP assay was monitored by real-time measurement of turbidity. (b) Sensitivity of LAMP for* T. asahii *detection was visualized by gel electrophoresis. The positive reaction was seen as a ladder-like pattern on 1% agarose gel electrophoresis analysis. (c) Sensitivity of PCR for* T. asahii *detection was visualized by gel electrophoresis. The positive reaction was seen as a ladder-like pattern on 1% agarose gel electrophoresis analysis. Marker, DL2000 DNA marker. (d) The specificity of LAMP for positive detection by naked eye detection. Green colour was observed using the naked eye in the tube which contained* T. asahii*, whereas others remained light orange after the reaction.

Tenfold serial dilutions of total genomic DNA from the* T. asahii* strain CBS2479 were used to test the sensitivity of the LAMP assay. The results indicated that the detection limit for the LAMP reaction was between 3.56 × 10^3^ and 3.56 × 10^2^ genome copies of template (Figures 3(a), 3(b), and 3(d)), while it was 3.56 × 104 genome copies for the PCR assay (Figure 3(c)). This suggested that the LAMP assay is 10 times more sensitive than the PCR assay.

### 3.4. Clinical Sample Detection

All samples from confirmed* T. asahii*-positive mice tested positive, whereas blood samples from unaffected mice tested negative (Table 2). After the patients with invasive trichosporonosis were proven by blood culture and the isolates were identified using the API 20C Aux yeast identification system, the blood samples were tested by the LAMP assay. The correlation between the LAMP assays and the culture and biochemical method results of the same tissue samples proved to be coincident.

## 4. Discussion

In this study, we reported on LAMP, a novel rapid detection method for identification of the pathogenic fungus* Trichosporon asahii*. An accurate and rapid diagnosis system for trichosporonosis caused by* T. asahii* is critical for early correct treatment. Trichosporonosis is a type of fungal infectious disease for which a series of PCR-based diagnostic systems has been developed [10]. Despite their simplicity and accuracy, PCR-based diagnostic methods are not widely used in clinics as routine diagnostic tools due to the need for a thermal cycler and the sequence analysis of DNA amplification products.

Compared with other PCR-based methods, LAMP is a powerful and innovative technique that provides a simple and rapid tool for early detection of infectious pathogens [22, 23]. A set of two inner primers and two outer primers are designed to recognize six distinct regions of the target DNA. The LAMP assay described in this paper is advantageous due to its simple operation, rapid reaction, and ease of detection. It can be conducted in 1 hour or less and is thus very fast. Even after including the time required to manipulate the sample and extract the DNA, the total assay duration is only approximately 3 hours. However, other common assays used in clinical identification, such as strain culturing and fungal identification by microscopy, the API 20 system, or rRNA gene PCR product sequencing, require 2 to 7 days to complete [16]. In contrast, the only equipment needed for the LAMP reaction is a standard laboratory water bath or a heating block that can provide a constant temperature.

In this study, we designed and compared additional primers based on different parts of IGS1 and used 15 different* T. asahii* strains, including different clinical strains isolated from Chinese patients, to confirm that our primers could accurately identify* T. asahii* clinical isolates. The LAMP primers in this study were designed from the intergenic spacer (IGS) 1 region of the rRNA gene. In contrast to ribosomal genes, which contain highly conserved sequences that are detected in close organisms, the intergenic spacer of rRNA (IGS) appears to be the most rapidly evolving spacer region. The IGS region displays higher polymorphism, which facilitates molecular identification. Both the conserved and the variable regions of IGS sequences are suitable for the development of PCR primers for pathogenic fungi. The data in this study show that our LAMP primer based on the IGS region is specific and could effectively identify* T. asahii* and differentiate it from other pathogenic fungi, especially other related pathogenic* Trichosporon* strains. Both Kohei and our study demonstrate the preferred choice of the IGS sequences for the molecular identification of closely related species.

To develop a detection system for* T. asahii*, we evaluated the sensitivity assay. LAMP amplifies DNA with high efficiency under isothermal conditions without a significant influence from the copresence of nontarget DNA. In this study, the LAMP method of detecting* T. asahii* was found to be highly sensitive, as it could detect levels of* T. asahii* up to 3.56∗10^3^ genome copies fungal DNA per reaction, whereas, by PCR, the detection of* T. asahii* was possible only up to 3.56 ∗ 10^4^ genome copies of fungal DNA per reaction. This indicates that the sensitivity of LAMP is ten times higher than that of standard PCR. This increased sensitivity makes LAMP a better choice than PCR for the detection of* T. asahii* in cases where lower fungal concentrations are expected. The LAMP assay also successfully detects the presence of* T. asahii* among clinical samples.

Additionally, the LAMP assay produces a large amount of amplified product, resulting in easier detection by visual observation of an increase in turbidity caused by generation of magnesium pyrophosphate. SYBR green was used as an indirect method for the detection of positive reactions. SYBR green has been shown to not affect the LAMP reaction and can thus be added to the master mix before the DNA amplification step, therefore avoiding the need to open the tubes at the end of the reaction [22]. This is an important advantage over other visualization methods because the LAMP products are an important source of cross contamination [24]. The change in turbidity can also be measured quantitatively and in real-time by using a real-time turbidimeter [22]. The amplified product could be detected by a conventional approach of gel electrophoresis, but this method is easily contaminated and leads to false positives. In this study, there is no difference among the three methods. However, according to our knowledge, a turbidimeter is the better way to measure the product.

In summary, in the current study, we showed that the LAMP technique based on the IGS region enables specific detection of* T. asahii* but excludes related* Trichosporon* species and other pathogenic fungi. It is a novel technique that can potentially be used for rapid diagnosis of* T. asahii* infections, not only in laboratories but also in an outpatient clinic setting. The method can be applied not only to cultures but also to a variety of clinical samples. This can be of great significance for detecting organisms that cause invasive or disseminated infections but are difficult to cultivate from the samples, such as the zygomycete species. In conclusion, the LAMP method described in this study represents a new, sensitive, specific, and rapid protocol for the detection of* T. asahii*.

## Figures and Tables

**Table 1:** Loop-mediated isothermal amplification (LAMP) detection of the strains used in this study.

Species	Strains or source
*Trichosporon asahii *	CBS 2479
*Trichosporon asahii *	CBS 8904
*Trichosporon asahii *	CBS 7137
*Trichosporon asahii *	BZ701, Clinical isolates, Hubei, China
*Trichosporon asahii *	BZ702, Clinical isolates, Hubei, China
*Trichosporon asahii *	BZ703, Clinical isolates, Beijing, China
*Trichosporon asahii *	BZ704, Clinical isolates, Guangdong, China
*Trichosporon asahii *	BZ705, Clinical isolates, Shandong, China
*Trichosporon asahii *	BZ706, Clinical isolates, Shanghai, China
*Trichosporon asahii *	BZ707, Clinical isolates, Shanghai, China
*Trichosporon asahii *	BZ708, Clinical isolates, Shanghai, China
*Trichosporon asahii *	BZ709, Clinical isolates, Shanghai, China
*Trichosporon asahii *	BZ710, Clinical isolates, Shanghai, China
*Trichosporon asahii *	BZ901, Clinical isolates, Chongqing, China
*Trichosporon asahii *	BZ902, Clinical isolates, Shanghai, China
*Trichosporon domesticum *	CBS 8280
*Trichosporon dermatis *	CBS 2043
*Trichosporon jirovecii *	CBS 6864
*Trichosporon mucoides *	CBS 7625
*Trichosporon asteroides *	CBS 2481
*Trichosporon coremiiforme *	CBS 2482
*Trichosporon inkin *	CBS 5585
*Trichosporon japonicum *	CBS 8641
*Trichosporon lactis *	CBS 9051
*Trichosporon ovoides *	CBS 7556
*Trichosporon dohaense *	CBS 10761
*Trichosporon faecale *	CBS 4828
*Trichosporon debeurumanianum *	CBS 1896
*Trichosporon montevideense *	CBS 8261
*Cryptococcus gattii *	S8012, Clinical isolates, Shanghai, China
*Cryptococcus laurentii *	CICC31237, CGMCC
*Cryptococcus luteolus *	ATCC10671
*Cryptococcus neoformans var. grubii *	H99, National Institutes of Health, USA
*Cryptococcus neoformans var. neoformans *	Clinical isolates, Shanghai, China
*Cryptococcus podzolicus *	CBS6819
*Cryptococcus podzolicus *	Clinical isolates, Shanghai, China
*Pichia pastoris *	Environmental isolates, Hubei, China
*Candida albicans *	ATCC10231
*Candida glabrata *	ATCC28226
*Candida krusei *	ATCC2159
*Candida parapsilosis *	ATCC90018
*Candida parapsilosis *	Clinical isolates, Shanghai, China
*Candida stellata *	ATCC10667
*Candida tropicalis *	ATCC66029
*Debaryomyces hansenii *	ATCC4144
*Fonsecaea pedrosoi *	SCZ10025
*Exophiala dermatitidis *	SCZ10002
*Filobasidiella neoformans var. neoformans *	CBS7815
*Sporothrix schenckii *	SCZ10142
*Acremonium chrysogenum *	ATCC20416
*Aspergillus fumigatus *	SCZ10130
*Aureobasidium pullulans *	Environmental isolates, Jiangsu, China

ATCC: American type culture collection, Rockville, MD, USA; CBS: Centraalbureau voor Schimmelcultures, Baarn, The Netherlands; SCZ: Shanghai Key Laboratory of Molecular Medical Mycology, Shanghai Changzheng Hospital, Shanghai, China; CGMCC: China General Microbiological Culture Collection Center, China.

**Table 2:** Sequences of LAMP primers used for detection of IGS1.

Primers name	Sequence (5′-3′)
TA-19F3	CTTGGTCTTTGCAGCTCCTA
TA-19B3	GGGAGACAAGAGGTCTCTGG
TA-19FIP	GAGGCTGAGGTCTCGATGTGATTTGTATGCTCACCGGTACAGAC
TA-19BIP	CCGCCTACCTCTGAGGCCTTTTTCAGGGCGGTTGAGGACTA
TA-19LF	AGAGCTGGCAGGCTTGG
TA-19LB	CTGTCCAAAGGGGCCTGGT

**Table 3:** Clinical specimens used in this study and the results of different detection methods.

Sample number	Source	LAMP	Culture	API 20 C AUX systems
1	Mouse, blood	+	+	
2	Mouse, blood	+	+	
3	Mouse, blood	+	+	
4	Mouse, blood	+	+	
5	Mouse, blood	+	+	
6	Mouse, urine	+	+	
7	Mouse, urine	+	+	
8	Mouse, urine	+	+	
9	Mouse, peritoneal irrigation fluid	+	+	
10	Mouse, peritoneal irrigation fluid	+	+	
11	Mouse, blood	−	−	
12	Mouse, blood	−	−	
13	Mouse, blood	−	−	
14	Mouse, blood	−	−	
15	Mouse, blood	−	−	
16	Human, blood	+		*T*. *asahii *
17	Human, blood	+		*T*. *asahii *
18	Human, blood	+		*T*. *asahii *
19	Human, blood	+		*T*. *asahii *
20	Human, blood	+		*T*. *asahii *
21	Human, blood	+		*T*. *asahii *
22	Human, blood	−		*C*. *albicans *
23	Human, blood	−		*C*. *albicans *
24	Human, blood	−		*C*. *tropicalis *
25	Human, blood	−		*C*. *albicans *
